# Enhanced Thermal Stability of Conductive Mercury Telluride Colloidal Quantum Dot Thin Films Using Atomic Layer Deposition

**DOI:** 10.3390/nano14161354

**Published:** 2024-08-16

**Authors:** Edward W. Malachosky, Matthew M. Ackerman, Liliana Stan

**Affiliations:** 1 QDIR, Inc., 3440 S. Dearborn St. Suite 114S, Chicago, IL 60616, USA; ewmalachosky@qdinfrared.com; 2 Argonne National Laboratory, Center for Nanoscale Materials, Lemont, IL 60439, USA; lstan@anl.gov

**Keywords:** atomic layer deposition, colloidal quantum dots, shortwave infrared, CQD, ALD, thermal stability, photodetector, mercury telluride, HgTe

## Abstract

Colloidal quantum dots (CQDs) are valuable for their potential applications in optoelectronic devices. However, they are susceptible to thermal degradation during processing and while in use. Mitigating thermally induced sintering, which leads to absorption spectrum broadening and undesirable changes to thin film electrical properties, is necessary for the reliable design and manufacture of CQD-based optoelectronics. Here, low-temperature metal–oxide atomic layer deposition (ALD) was investigated as a method for mitigating sintering while preserving the optoelectronic properties of mercury telluride (HgTe) CQD films. ALD-coated films are subjected to temperatures up to 160 °C for up to 5 h and alumina (Al_2_O_3_) is found to be most effective at preserving the optical properties, demonstrating the feasibility of metal–oxide in-filling to protect against sintering. HgTe CQD film electrical properties were investigated before and after alumina ALD in-filling, which was found to increase the p-type doping and hole mobility of the films. The magnitude of these effects depended on the conditions used to prepare the HgTe CQDs. With further investigation into the interaction effects of CQD and ALD process factors, these results may be used to guide the design of CQD–ALD materials for their practical integration into useful optoelectronic devices.

## 1. Introduction

Colloidal quantum dots (CQDs) are of great interest for optoelectronics including light emitting devices [1], energy-harvesting photovoltaics [2], solar concentrators [3], and photodetectors [4]. However, when subjected to the elevated temperatures that can occur during manufacturing, testing, or while in use, CQDs and their device performance can degrade. One mode of degradation is thermal sintering, during which neighboring CQDs fuse together. Sintering occurs at elevated temperatures as the activation barrier to ion diffusion is overcome and interfaces can reconstruct to reduce the high surface energy of the CQDs. Consequently, quantum confinement is lost during sintering, causing broadening of the absorption spectrum and harmful changes to the electrical properties of the films.

One solution to this problem is to grow a shell on the surface of the CQDs to prevent neighboring core materials from fusing together. By selection of the appropriate shell material, one or both of the charge carriers in the core CQD can be localized to either the core or the shell of the material. Such core–shell structures where both carriers are confined to the core CQD have been most successful for light emitting and display technologies. A core/shell that efficiently separates one charge carrier from the core to the shell may be used to separate charge carriers in photovoltaic devices, as was recently demonstrated by Peng et al. [5] For narrow-band-gap CQDs, like HgTe, where appropriate materials for band engineering may not be readily available, a sufficiently thin shell—i.e., a single monolayer of material—may be enough to promote stability while allowing for charge carriers to be extracted from the core CQD under an applied electric field, as Zhang et al. have recently reported with HgTe/CdS CQDs in a photoconductive infrared imaging array [6]. However, even in this case the use of the thin shell still resulted in a 100-times lower carrier mobility and the electrical properties of the HgTe/CdS core/shells were susceptible to mild heating (up to 100 °C) for short periods of time (<1 h). HgTe CQDs are especially relevant as an emerging low-cost and scalable nanomaterial replacement for more expensive epitaxial HgCdTe and III-V type-II superlattice detectors useful in surveillance and thermal infrared imaging applications beyond silicon [7]. Therefore, a method other than a shell is needed that can preserve the optical and electrical properties of CQD thin films for the practical development of CQD-based, charge-collecting devices.

In bulk semiconductor devices, metal oxides and nitrides have been used to protect the integrity of the active elements and interconnects from metal ion diffusion at elevated process temperatures and during operation [8]. Such diffusion barriers may also prevent sintering between neighboring colloidal quantum dots without preventing charge transport, if such a diffusion barrier could be grown within the CQD thin film. Low-temperature atomic layer deposition (ALD) has been used to fill the spaces in CQD films with metal oxides, leading to improved charge carrier transport and providing protection against oxidation for air-stable PbX (X = S,Se) [9,10] and InP CQDs [11]. However, the impact of in-filled ALD metal oxides on the thermal stability of CQD solids has not yet been reported.

Here, metal oxides as barriers were prepared by low-temperature ALD on conductive HgTe CQD solid films. Zinc oxide (ZnO), titanium dioxide (TiO_2_), and alumina (Al_2_O_3_) were readily available and evaluated as barrier materials against thermally induced sintering. The optical and electronic properties were evaluated to determine the effectiveness of the respective barrier material against sintering before and after subjecting the ALD-coated HgTe CQD thin films to elevated temperatures.

## 2. Materials and Methods

### 2.1. Preparation of Trioctylphosphine Telluride (TOPTe)

A stock solution of 1 M tellurium precursor was prepared by dissolving a tellurium shot in trioctylphosphine (TOP) at 100 °C overnight in a nitrogen-filled glovebox.

### 2.2. Synthesis and Post-Synthesis Processing of p-Type HgTe Colloidal Quantum Dots

For p-type HgTe colloidal quantum dots, the procedure was adapted from Ackerman et al. [12]. In a typical synthesis, 54 mg (0.1 mmol) of HgCl_2_ and 10 mL oleylamine were transferred to a 50 mL round-bottom flask. The reaction solution was left to mix at 100 °C under a nitrogen flow for 1 h. The reaction temperature was reduced to 85 °C. Then, 0.2 mL of TOPTe was diluted in 10 mL oleylamine (10 mL), and the mixture was rapidly injected into the round-bottom flask. The reaction was allowed to proceed for 1-to-10 min before quenching with 10 mL of tetrachloroethylene and cooling to room temperature. A 1 mL addition of 1-dodecanethiol was added to the solution, followed by precipitation once with ethanol. The supernatant was discarded, and the pellet dissolved in 10 mL of chlorobenzene. A white gel was present on the surface of the solution, and a 1 mL fraction of TOP was added to remove this gel. The solution was again precipitated with ethanol and re-dissolved in chlorobenzene. No white gel was observed after the addition of TOP and the second precipitation. The solution was stored in ambient atmosphere and temperature until further use.

HgTe CQDs were cleaned with dodecylethyldimethylammonimum bromide (DEDAB) (0.1 M in isopropanol) and isopropanol (IPA), and then dissolved in a mixture of chlorobenzene and butyl acetate. Thin films were drop-casted at 40 °C under ambient conditions onto sapphire substrates for thin film absorption measurements or on interdigitated electrodes for electrical measurements. Thin films were then crosslinked for 30 s by soaking in an ethanedithiol (1%)/hydrochloric acid (1%)/IPA solution, followed by rinsing with neat IPA and blown dry with compressed air.

### 2.3. Synthesis and Post-Synthesis Processing of n-Type HgTe Colloidal Quantum Dots

For n-type HgTe colloidal quantum dots, the procedure was adapted from Yang et al. [13]. In a typical synthesis, 180 mg (0.66 mmol) of HgCl_2_ and 10 mL of oleylamine were transferred to a 50 mL round-bottom flask. The mixture was heated at 100 °C for 1 h under vacuum to fully dissolve HgCl_2_. The temperature was reduced to 80 °C, the vacuum was closed, and the nitrogen flow was opened for the reaction. Then, a premixed solution of 200 μL of TOPTe, 50 μL of 1-dodecanethiol, and 800 µL of oleylamine was quickly injected into the HgCl_2_ solution. After 1 min, a second premixed solution of 200 μL of TOPTe in 800 μL of oleylamine was added dropwise over 2 min. The reaction proceeded for another 3 min and then was quenched with 10 mL of tetrachloroethylene. The heating mantle was replaced with a water bath to further cool to room temperature. At a temperature less than 30 °C, 0.6 mL of 3-mercaptopropionic acid was injected and left to mix under nitrogen for 1 h. The HgTe CQDs were then precipitated with isopropanol. The clear supernatant was discarded and the HgTe CQDs were stored in octane in a nitrogen glovebox until further use.

To prepare the n-type HgTe CQDs for coating, a phase transfer process was used to control the n-type doping. Typically, 180 mg (0.66 mmol) HgCl_2_, 140 μL of 2-mercaptoethanol, and 400 μL of n-butylamine were pre-dissolved in 5 mL of dimethylformamide (DMF). Then, 1 mL of the DMF solution was transferred to a centrifuge tube along with 1 mL of the HgTe CQD in octane stock solution. The phases were mixed for 1 min and allowed to separate, after which the HgTe CQDs had transferred to the DMF phase. The octane phase was removed, followed by washing 3 times with 1 mL of neat octane to residual non-polar materials. Once the last octane washing phase was removed, HgTe CQDs in DMF were precipitated by the addition of 1 mL of toluene. The clear supernatant was discarded and the HgTe CQDs were redispersed with 200 μL of DMF to obtain concentrated ink. The ink was blade coated at 60 °C onto sapphire substrates for FTIR measurements or interdigitated electrodes on doped silicon substrates for conductance and FET measurements. Thin films were then crosslinked for 30 s by soaking in an ethanedithiol (1%)/hydrochloric acid (1%)/IPA solution, followed by rinsing with neat IPA and blown dry with compressed air.

### 2.4. Atomic Layer Deposition on Colloidal Quantum Dot Thin Films

The ALD infill of HgTe CQD films presented in this paper was performed using an Arradiance Gemstar benchtop system. The metal precursors used were trimethylaluminum (TMA) for Al_2_O_3_, diethyl zinc (DEZ) for ZnO, and tetrakis(dimethylamino)titanium (TDMAT) for TiO_2_. Water was used as an oxidant for all films. High-exposure-mode ALD processes that had two half cycles were used. A half cycle consisted of a precursor injection in a series of short pulses, followed by an exposure time, and then a purging step. During the high-exposure-mode ALD process, the evacuation valve was closed before introducing the precursor in the chamber and opened after the exposure time. N2 was used as a carrier and purging gas. The experimental parameters are listed below (Table 1).

### 2.5. Characterization of Optical Properties

Absorption spectra of HgTe colloidal quantum dot thin films were made using a Nicolet 550 FTIR. Samples were measured in transmission mode. The sample, consisting of a HgTe CQD thin film (with and without ALD) on a sapphire substrate, was placed in the beam path in the sample chamber such that the sapphire surface was oriented with a 45° tilt with respect to the incident beam path. The sample tilt was necessary to minimize interference effects—due to the different indices of the CQD and sapphire substrate in the optical path—while preserving the lineshape of the CQD thin film absorption.

### 2.6. Conductance and Field-Effect Transistor Measurements

Conductance and field-effect transistor (FET) measurements were made using substrates consisting of Au-Au interdigitated electrodes on a doped silicon/silicon dioxide substrate. Gold (Au) interdigitated electrodes (30 pairs: each pair have a 5 µm channel width and 1 mm finger length) were patterned by photolithography and the e-beam deposition of Ti(20 nm)/Au(80 nm) on the n++ doped silicon substrate with a 300 nm silicon dioxide surface layer. The doped silicon substrate and 300 nm silicon dioxide layers served as the backside gate and insulator, respectively, in the thin film field-effect transistor. For conductance measurements, the silicon backside gate was left open and electrically insulated from the surface conductor. For FET measurements, a 1 V bias was applied between the source and drain interdigitated contacts, while a variable gate bias (V_g_) was applied to the silicon backside gate. The conductor or FET samples were placed on the probe station stage and aluminum wire probes were brought into electrical contact with the source, drain, and gate contacts. A Keithley 4200 parameter analyzer was used to program the source, drain, and gate biases and to measure the conductor current or FET source–drain current as a function of gate voltage, respectively. All measurements were carried out at room temperature under ambient conditions.

## 3. Results and Discussion

Thin films of HgTe CQDs were coated onto sapphire substrates and in-filled by ALD with different metal oxides. Infrared–transparent sapphire was selected as the substrate to facilitate FTIR absorption measurements before and after ALD and high-temperature baking. The amount of the metal oxide deposited was controlled by the number of cycles and dwell time of the precursor gases.

**Figure 1:**
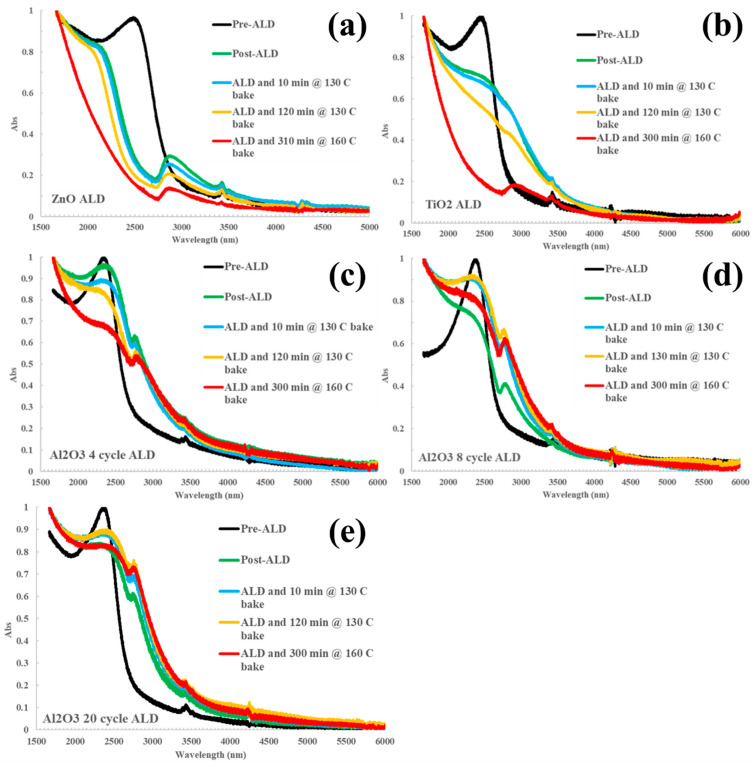
Infrared absorption spectra plotted against the wavelength (nm) of HgTe CQD thin films on sapphire substrates measured as a function of atomic layer deposition cycles and bake conditions. Evolution of the infrared absorption spectrum for HgTe CQD after (**a**) ZnO, (**b**) TiO_2_, (**c**) 4 cycles of alumina, (**d**) 8 cycles of alumina, and (**e**) 20 cycles of alumina and being subjected to baking up to 165 °C for up to 5 h.

Figure 1 shows the evolution of the HgTe CQD infrared absorption spectra as a function of ALD deposition and baking conditions for zinc oxide (ZnO), titanium dioxide (TiO_2_), and alumina (Al_2_O_3_). Both ZnO and TiO_2_ failed to preserve the exciton absorption feature following ALD such that these materials were deemed to be incompatible with HgTe CQDs as a barrier against thermal sintering. In the case of ZnO (Figure 1a), the exciton absorption feature of the HgTe CQDs blue-shifted immediately upon ALD treatment. The spectral shape was maintained with ZnO coating until the baking was increased to 160 °C for 5 h, at which point the exciton absorption feature was completely quenched. For TiO_2_ (Figure 1b), the exciton feature broadened and red-shifted following ALD, then failed to mitigate sintering when baked above 130 °C, as evidenced by the loss of the exciton feature. The additional peaks found between 2700 and 3000 nm can be assigned to coordinated hydroxyl group residuals at the surface of the ALD-deposited oxides, varying in position depending on coordination number and species with respect to Al, Zn, or Ti [14,15,16].

In contrast, Al_2_O_3_ both preserved the exciton feature overall—suffering a marginal broadening and red-shift—and provided significantly improved thermal stability against sintering compared to ZnO and TiO_2_. Figure 1c–e show the evolution of the absorption spectrum with increasing the temperature and increasing the number of cycles (4, 8, and 20, respectively) of the alumina on the HgTe CQD. A minor red-shift was observed between the control and post-ALD/pre-bake steps that cannot be attributed to thermal effects. Between 8 cycles (Figure 1d) and up to 20 cycles (Figure 1e) of ALD were sufficient to retain the exciton feature of the absorption spectrum after baking at 130 °C for 2 h and 160 °C for 5 h. This demonstration is evidence that ALD can be used to fabricate barriers that can provide thermal stability against sintering. Further development of alumina—as well as the investigation of other materials such as metal chalcogenides that are commonly used with infrared semiconductors—may lead to the robust design of CQD-based optoelectronics, especially photodetectors.

**Figure 2:**
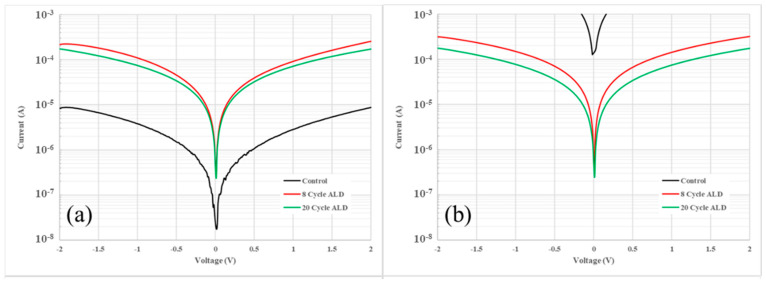
Log plots of the current versus voltage for HgTe CQD thin film Au-Au photoconductor devices with a 5-microns electrode gap. The conductance for a control device (black) that was not subjected to atomic layer deposition, a film subjected to 8 cycles of alumina ALD (red), and a film subjected to 20 cycles alumina ALD (green) are plotted for comparison. Conductance of HgTe CQD films measured (**a**) before and (**b**) after baking at 130 °C for 2 h under a nitrogen environment are shown.

Having established the feasibility of ALD as a method for preserving the integrity of the HgTe CQDs, the impact of ALD on the electrical properties of these thin films was investigated. First, simple photoconductor measurements, as shown in Figure 2, were used to assess the impact of ALD in-filling on the conductance of HgTe CQD films. Thin films of HgTe CQDs were deposited on gold interdigitated electrodes and then coated with either no alumina (control sample), 8 cycles, or 20 cycles of alumina by ALD. The photoconductors were characterized before and after baking at 130 °C for 2 h. Comparing current levels before baking (Figure 2a) and after baking (Figure 2b), it was apparent that both 8 and 20 cycle alumina ALD preserved the conducting properties of the HgTe CQD film, while un-passivated films degraded, as indicated by the significant increase in current after baking.

An order-of-magnitude increase in the conductance following ALD coating was also notable, as shown in Figure 2a, before subjecting the films to baking. An increase in the conductance of the CQD film could follow from a higher carrier mobility, higher doping density, or both due to ALD in-filling. To investigate what contributed to this change, field-effect transistor (FET) measurements were made on HgTe CQD thin films before and after ALD. Samples consisted of HgTe CQD films coated on Au-Au interdigitated electrodes (source and drain) patterned on a heavy n-type doped silicon substrate (gate) with a 300 nm SiO_2_ dielectric layer. Measurements were made across multiple FET devices.

**Figure 3:**
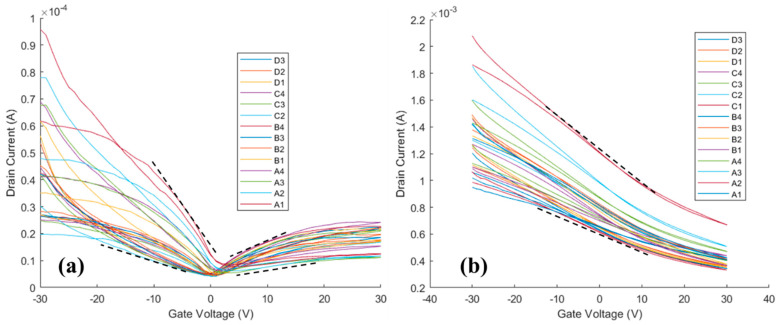
Transfer curves for HgTe colloidal quantum dot thin films measured at a 1 V source–drain bias (**a**) before alumina ALD and (**b**) after alumina ALD. Black dashed lines indicate the maximum and minimum slopes taken to calculate the carrier mobilities.

The transfer curves, shown in Figure 3, were measured before (Figure 3a) and after (Figure 3b) alumina ALD and analyzed to understand the impact of ALD on the mobility and doping of the HgTe CQD thin films. Before ALD (Figure 3a), the average minima of conductance were at gate voltages (V_g_) between 0 V and +5 V, which suggests light p-type doping of the thin films. After ALD (Figure 3b), the minima of conductance were greater than +30 V and not observable on the scale of the measurement, suggesting a shift to heavy p-type doping of the thin films following ALD coating.

With respect to carrier transport, the carrier mobilities were estimated by taking the slopes indicated by the dashed lines in Figure 3 and calculating the mobility (*µ_FET_*) according to the expression $\mu_{FET}=\frac{L}{C_{i}V_{SD}W}\frac{dI_{SD}}{{dV}_{g}}$, where *L* is the channel length (gap), *W* is the channel width, *V_SD_* is the source–drain bias, *C_i_* is the capacitance per unit area for the 300 nm SiO_2_ insulator, *I_SD_* is the source–drain current, and *V_g_* is the gate voltage [17]. Before ALD (Figure 3a), carrier mobilities were 5 × 10^−3^ up to 4 × 10^−2^ cm^2^/Vs for holes and 10^−3^ up to 10^−2^ cm^2^/Vs for electrons. However, after ALD (Figure 3b) the FET was dominated by hole currents with hole mobilities from 0.2 to 0.4 cm^2^/Vs: a 10-times increase in hole mobility following alumina ALD. Both the increase in the doping and the increase in the carrier mobility contribute to the increased conductance of the HgTe CQD thin films following ALD.

From these observations, it is reasonable to conclude that alumina ALD coatings will consistently increase the p-type doping and carrier mobilities of HgTe CQD thin films. Compared to HgTe CQDs, prior works on Pb chalcogenides have higher carrier mobilities because of ALD in-filling, attributing the improvements to trap filling for alumina-coated Pb chalcogenides [10]. However, with respect to doping, Pb chalcogenides consistently showed less p-type and more n-type doping: that is, the opposite direction to the changes observed here for HgTe CQDs. The changes in doping for Pb chalcogenides have been attributed to acceptor state passivation by alumina ALD, thus reducing the p-type doping density [10]. By analogy, then, alumina ALD on HgTe CQDs could have the effect of passivating hole traps, increasing acceptor states, and/or decreasing donor states. Alternatively, changes in the net surface dipole of the CQDs following from reactions with the ALD precursors during deposition may also explain the doping shifts of these materials [18]. From the perspective of the CQDs, these effects are functionally equivalent, causing the Fermi level to shift relative to the conduction and valence levels and shifting the doping of the system.

To better understand the possible impact of ALD on the doping and mobilities of HgTe CQDs, n-type HgTe CQDs were synthesized as reported by Yang et al. and studied by FET measurements [13]. The n-type doping was achieved by transferring the HgTe CQDs from non-polar octane into polar N,N-dimethylformamide (DMF) in the presence of excess mercury chloride (HgCl_2_). The HgTe CQDs were cleaned to remove excess polar ligands and re-dissolved in DMF. Thin-film backside-gated FETs were made using n-type HgTe CQDs, which were coated from DMF, to characterize their doping and mobility before and after ALD in-filling. From the previous discussion, it was reasonable to expect that the n-type HgTe CQDs would become increasingly p-type and the carrier mobilities would improve with ALD in-filling.

**Figure 4:**
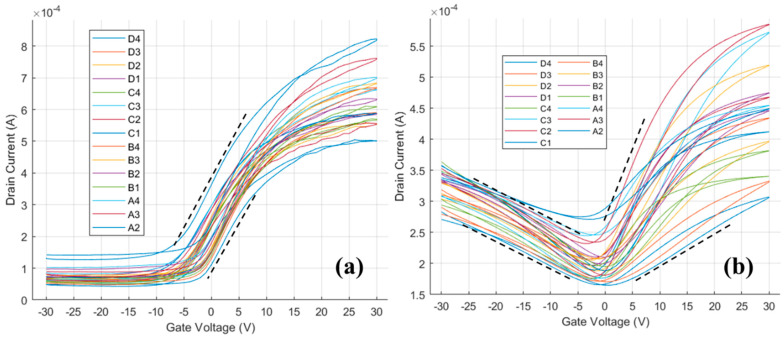
Transfer curves for n-type doped HgTe colloidal quantum dot thin films measured at a 1 V source–drain bias (**a**) before alumina ALD and (**b**) after alumina ALD. Black dashed lines indicate the maximum and minimum slopes taken to calculate the carrier mobilities.

The transfer curve for the n-type HgTe CQD film without and with ALD is shown in Figure 4. In this case, before ALD (Figure 4a) the HgTe CQDs show only electron transport with a carrier mobility of 0.3-to-0.5 cm^2^/Vs and minima of conductance at a V_g_ less than −5 V, which suggests lightly n-type doped films. Following ALD (Figure 4b), the minima of conductance shifted toward V_g_ = 0 V and hole currents were now measurable at negative gate voltages. Electron and hole mobilities following ALD were both between 0.1 and 0.3 cm^2^/Vs and comparable in magnitude to the electron carrier mobility before ALD. The shift in the conductance minima and the increase in the hole conductance were both consistent with a decreasing n-type character of the film following alumina ALD in-filling.

As in the previous case of p-type HgTe CQDs shown in Figure 3, ALD in-filling tended to decrease the n-type doping and increase the p-type doping of the HgTe CQD thin films. Contrary to the previous case however, the carrier mobilities were not improved, as evidenced by the comparable electron mobilities before and after ALD for the n-type HgTe CQDs. Furthermore, when p-type HgTe CQDs were prepared by phase exchange to polar DMF in a deficit of HgCl_2_ and then coated with alumina by ALD, similar results were observed (Supplementary Figure S3), with hole mobility effectively constant before and after alumina ALD. The impact of alumina ALD on carrier mobility was much less significant when HgTe CQDs were phase transferred to DMF. These observations may be explained by the differences in the processing of each of the HgTe CQD thin films investigated here.

## 4. Conclusions

In conclusion, low-temperature alumina (Al_2_O_3_) atomic layer deposition (ALD) was demonstrated as a feasible approach to prepare conductive films of HgTe colloidal quantum dots (CQDs) with an enhanced thermal stability against sintering. The thermal stability of the HgTe CQD thin films was established over the temperature range of 80 °C to 160 °C, with minimal impact on its optical properties and conductance of thin films before and after baking. Field-effect transistor (FET) measurements were used to study the impact of ALD on the electronic properties of the HgTe CQD films. Results from the FET measurements revealed that (1) carrier mobility was either preserved or improved and (2) doping generally shifts with a decreasing n-type and increasing p-type character due to alumina ALD in-filling. The magnitude of these effects following ALD was found to be dependent on the process conditions used to prepare the HgTe CQD films. While these investigations into the electronic properties were not exhaustive, the results reported here motivate the need for a better understanding of the interaction effects between the CQD structure, surface chemistry, and ALD materials. Under optimized conditions, then, it may be possible to engineer heterojunction and homojunction devices that are made thermally robust by ALD in-filling and passivation. A detailed understanding of how these factors interact and influence the electronic properties of CQD–ALD composites will be beneficial to engineer and manufacture robust CQD-based optoelectronic devices.

## Figures and Tables

**Table 1:** Experimental parameters for atomic layer deposition of metal oxides.

	ALD Material	Al_2_O_3_	ZnO	TiO_2_
	**Metal precursor**	TMA	DEZ	TDMAT
	**Reactant**	Water	Water	Water
	**Temperature**	80 °C	80 °C	80 °C
**First half cycle (metal precursor)**	**N2 flow**	5 sccm	10 sccm	10 sccm
	**Metal precursor pulse time**	150 ms	80 ms	700 ms
	**Pulse delay**	10 s	10 s	10 s
	**Number of pulses**	6	6	6
	**Exposure time**	600, 1200 s	400 s	120 s
	**Purge time**	90 s	90 s	240, 480 s
**Second half cycle (water)**	**N2 flow**	5 sccm	10 sccm	5 sccm
	**Water pulse time**	80 ms	80 ms	80 ms
	**Water pulse delay**	10 s	10 s	10 s
	**Number of water pulses**	6	6	6
	**Exposure time**	400, 800 s	400 s	240 s
	**Purge time**	120 s	120 s	240, 480 s
	**Number of cycles**	4, 8, 20	8	20

## Data Availability

The original contributions presented in this study are included in the article/Supplementary Materials; further inquiries can be directed to the corresponding author.

## Supplementary Materials

The following supporting information can be downloaded at https://www.mdpi.com/article/10.3390/nano14161354/s1: Figure S1: Temperature dependence of transfer curves for aggregated p-type HgTe CQD films through ALD coating and bakes; Figure S2: Absorption spectra of n-type HgTe CQDs before and after ALD, before and after baking; Figure S3: Temperature dependence and transfer curves for phase-transferred, p-type HgTe CQDs; Figure S4: Temperature dependence of transfer curves for phase-transferred, n-type HgTe CQDs.
