# Supplementary material for: Enhanced Thermal Stability of Conductive Mercury Telluride Colloidal Quantum Dot Thin Films Using Atomic Layer Deposition
Source: Nanomaterials (Basel). 2024 Aug 16;14(16):1354. doi: 10.3390/nano14161354 (PMC11357074; doi:10.3390/nano14161354)
Supplement: Supplementary file 1 [file nanomaterials-14-01354-s001.zip › nanomaterials-3132798-supplementary.pdf]

Supplementary Information:

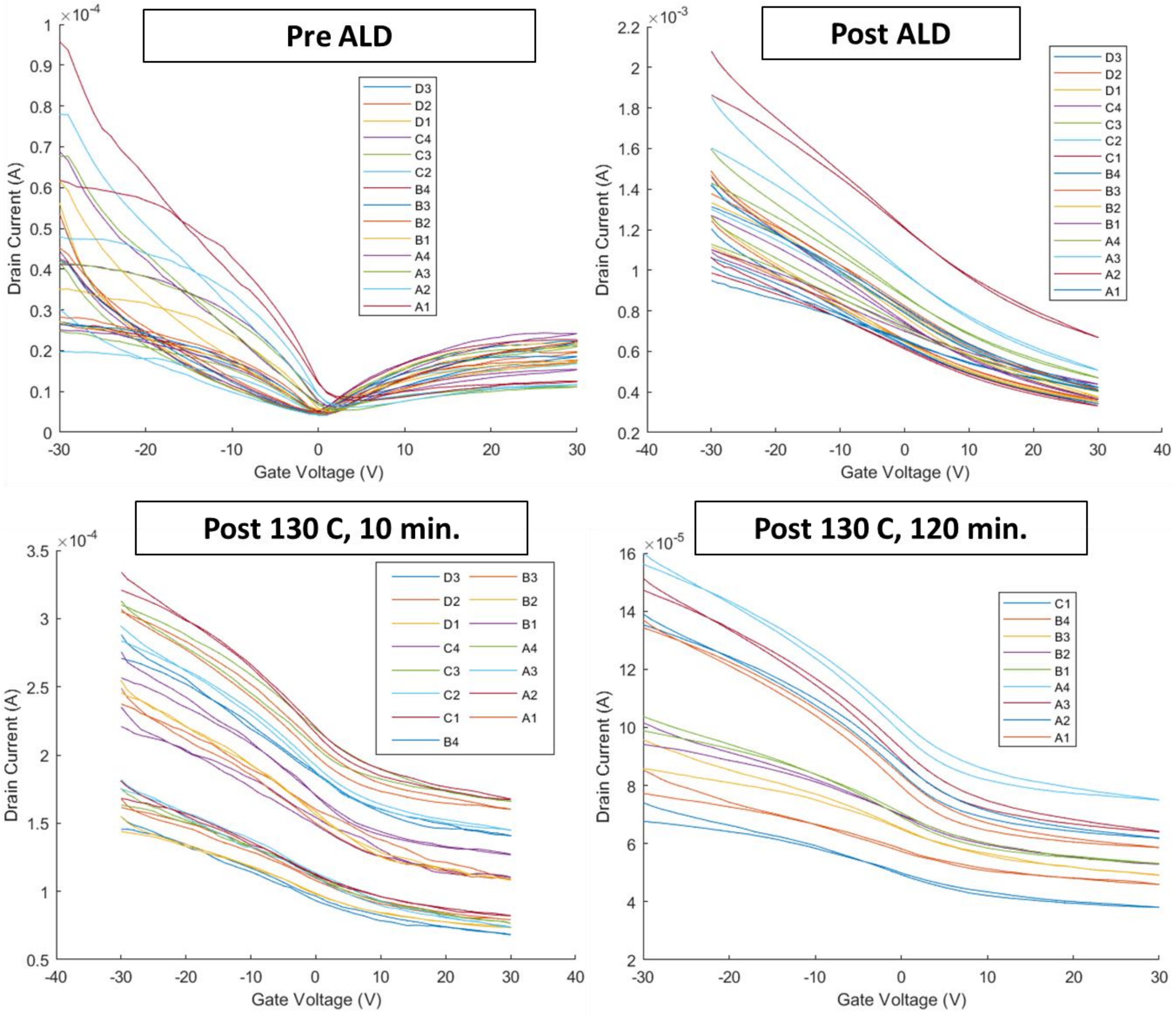

Figure S1. Temperature dependence of transfer curves for aggregated p-type HgTe CQD films through ALD coating and bakes. As bake times increase, current levels through the CQD films reduce by almost 15x, to ~2x the values observed in the original films.

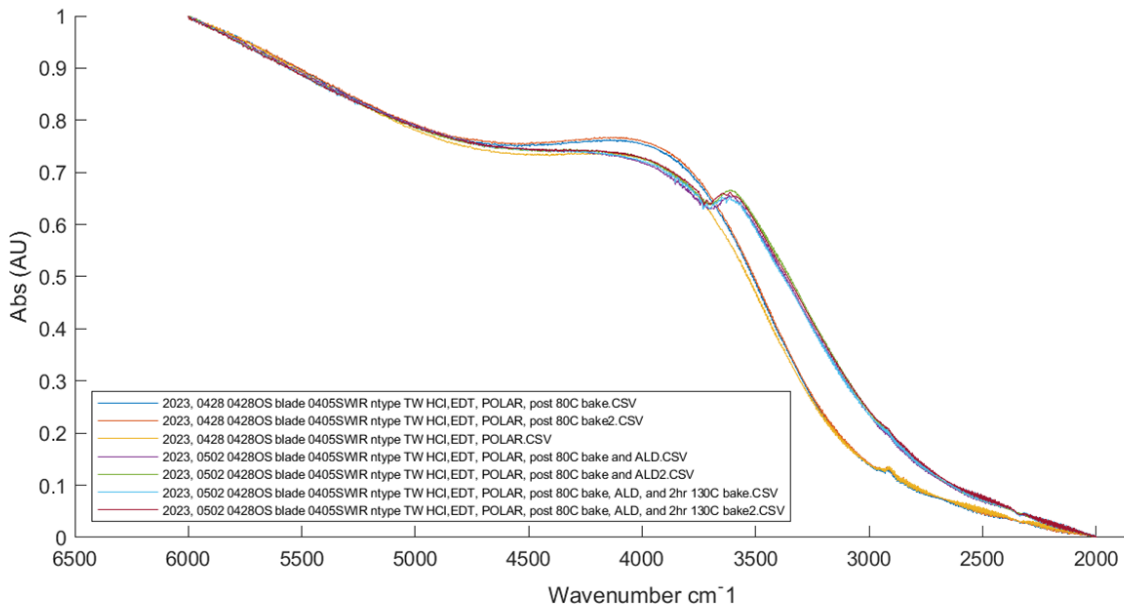

Figure S2. Absorption spectra of n-type HgTe CQD before and after ALD, before and after baking. No change in spectra is observed through the baking process.

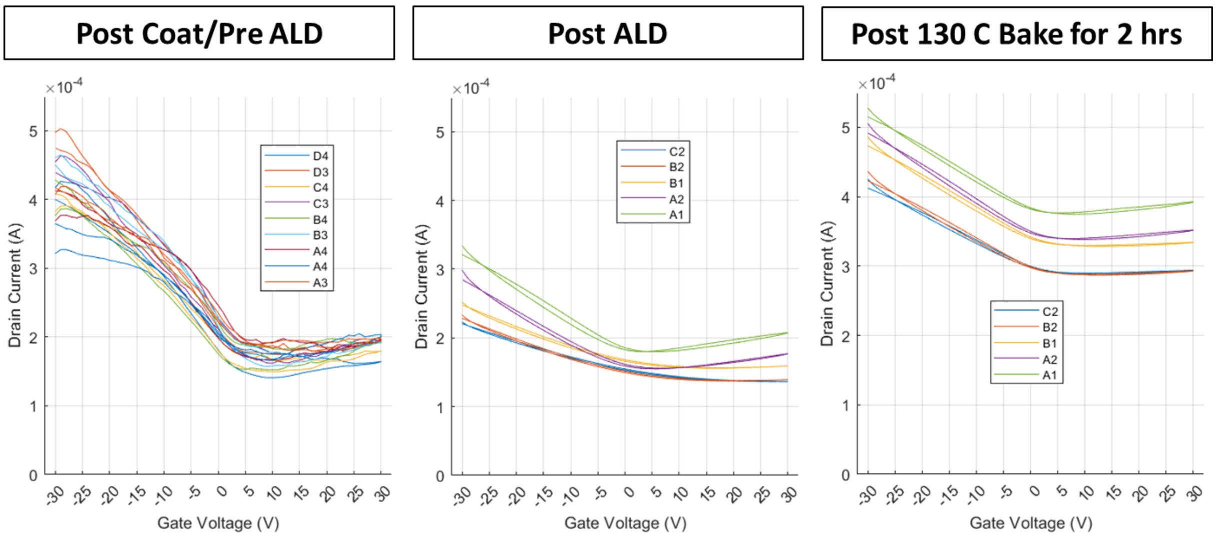

Figure S3. Temperature dependence and transfer curves for phase transferred, p-type HgTe CQD, synthesized according to Yang et al. Baking appears to increase conductivity through the film.

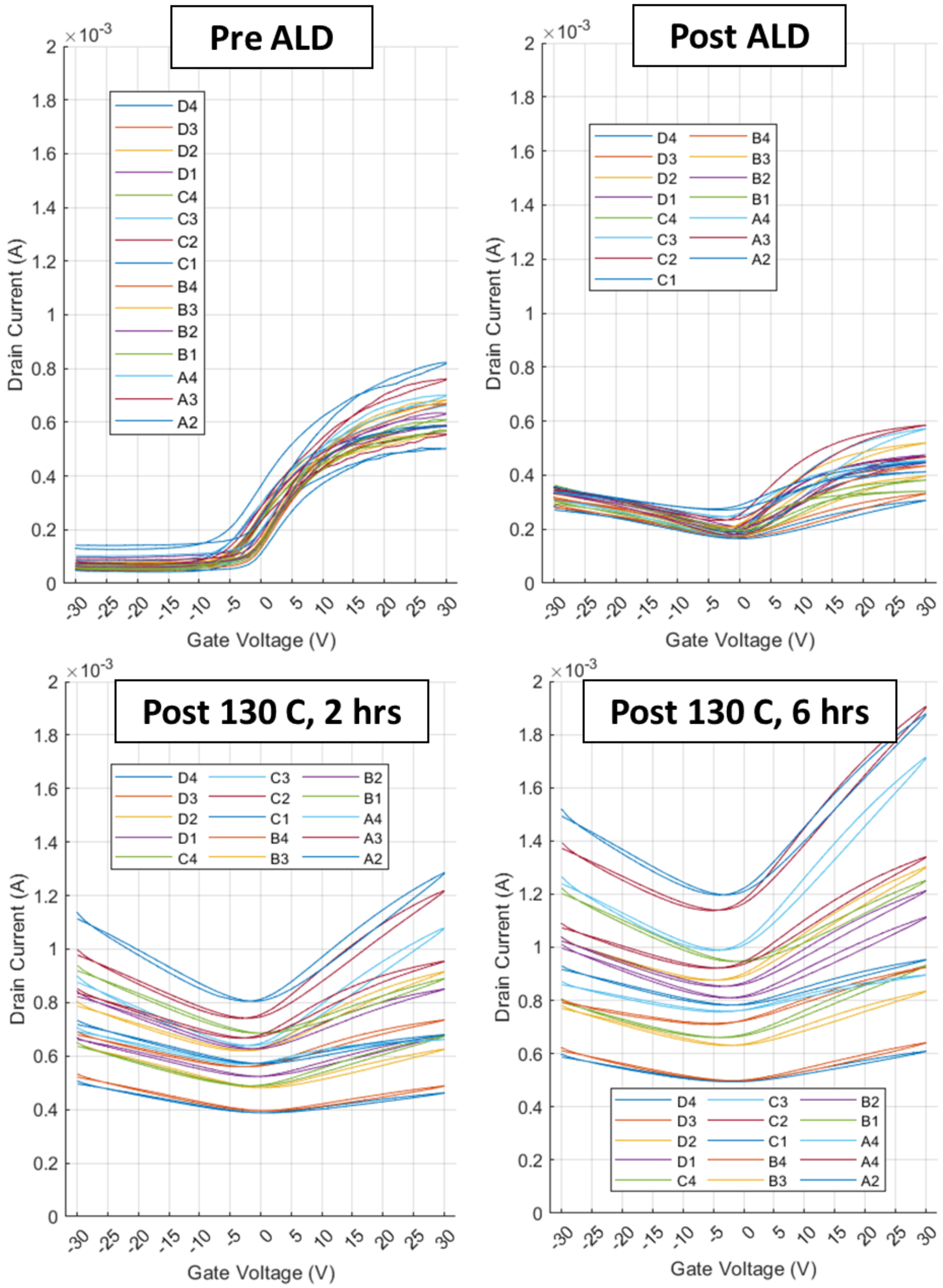

Figure S4. Temperature dependence of transfer curves for phase transferred, n-type HgTe CQD, synthesized according to Yang et al. As bake times increased, current value through the films also increased.
